# Tanshinol Attenuates the Deleterious Effects of Oxidative Stress on Osteoblastic Differentiation via Wnt/FoxO3a Signaling

**DOI:** 10.1155/2013/351895

**Published:** 2013-12-31

**Authors:** Yajun Yang, Yanjie Su, Dongtao Wang, Yahui Chen, Tie Wu, Gang Li, Xuegang Sun, Liao Cui

**Affiliations:** ^1^The Key Laboratory of Molecular Biology, State Administration of Traditional Chinese Medicine, School of Traditional Chinese Medicine, Southern Medical University, Guangzhou 510515, China; ^2^Department of Pharmacology, Guangdong Key Laboratory for R&D of Natural Drug, Guangdong Medical College, Zhanjiang 524023, China; ^3^Department of Orthopaedics and Traumatology, Faculty of Medicine, the Chinese University of Hong Kong, Hong Kong

## Abstract

There is now increasing evidence which suggests a pivotal role for oxidative stress in the development and progression of osteoporosis. We confirm herein the protective effects of natural antioxidant Tanshinol against oxidative stress in osteoblastic differentiation and the underlying mechanism. Our results show that hydrogen peroxide (H_2_O_2_) leads to accumulation of reactive oxygen species (ROS), decrease in cell viability, cell cycle arrest and apoptosis in a caspase-3-dependent manner, and inhibition of osteoblastic differentiation. Tanshinol reverses these deleterious consequence triggered by oxidative stress. Moreover, under the condition of oxidative stress, Tanshinol suppresses the activation of FoxO3a transcription factor and expressions of its target genes *Gadd45a* and *catalase (CAT)* and simultaneously counteracts the inhibition of Wnt signalling and expressions of target genes *Axin2*, *alkaline phosphatase (ALP)*, and *Osteoprotegerin (OPG)*. The findings are further consolidated using FoxO3a siRNA interference and overexpression of Tcf4. The results illustrate that Tanshinol attenuates oxidative stress via down-regulation of FoxO3a signaling, and rescues the decrease of osteoblastic differentiation through upregulation of Wnt signal under oxidative stress. The present findings suggest that the beneficial effects of Tanshinol may be adopted as a novel therapeutic approach in recently recognized conditions of niche targeting osteoporosis.

## 1. Introduction

Extensive evidence indicates that “oxidative stress” theory has currently been proposed as a new intensive mechanism that the development and progression of osteoporosis are in connection with an increase of oxidative stress, which leads to degenerative disease of bone tissue [1, 2]. Understanding of oxidative stress is currently a substantial topic of investigation with the aim of developing new ways to diminish negative effects of oxidative damage on bone metabolism [3]. Starting in their mid-40s, both genders experience a progressive decrease in bone mass and bone quality [2], associated with aging [4], some illnesses, or use of medications, such as diabetes or excessive exposure to glucocorticoids [5–7]. Notwithstanding, as for the evidence for physiological levels of reactive oxygen species (ROS) helpful in sustaining cellular function [8], it is generally believed that excessive accumulation of ROS causes oxidative stress and destroys proteins, lipids, and DNA, consequentially leading to cell death [9]. Based on current understanding of the cellular events of bone formation phase in the process of bone remodeling, increasing numbers of investigators have unraveled the potential mechanisms by which oxidative stress elicits a series of deleterious events in osteoblasts and ultimately contributes to osteoporosis [10, 11]. A sufficient number of mature osteoblastic cells are mainly required by bone formation during an episode of bone remodeling. The vast majority of Mesenchymal or stromal stem cells, however, fail to differentiate into osteoblast lineage cells by virtue of undergoing apoptosis under the condition of oxidative stress, which is responsible for the impairment of bone formation [12, 13]. Meanwhile, oxidative stress can also cause the increase in osteoclasts activity and upregulation of osteoclast differentiation [14, 15]. Therefore, a widely accepted participant in the pathogenesis of osteoporosis is oxidative stress, which causes a progressive increase in the prevalence of osteoblasts apoptosis, as well as an overwhelming decrease in the number of osteoblasts and bone formation rate. To sum up, impaired balance of bone remodeling may substantially contribute to bone loss under oxidative stress.

Cells neutralize the deleterious effects of ROS by up-regulating enzymatic scavengers like Catalase (CAT), and simultaneously by inducing DNA-damage repair genes such as *Gadd45a*, as well as several proapoptotic genes for the elimination of damaged or abnormal cells [3]. The response involves a series of molecular events initiated by activation of Forkhead box O (FoxO) transcription factors, which acts as an important defense mechanism against oxidative stress [11]. Mammalian FoxOs consist of four molecules, including FoxO1, FoxO3a, FoxO4, and FoxO6, which participate in the modulation of cellular proliferation, differentiation, and longevity in a variety of cells [3]. Several types of remolding-related cells in the bone tissue express FoxO1-3, of which FoxO3 is the predominant one, whereas FoxO6 is restricted to the developing brain [16, 17]. Activation of FoxO3a transcription factor by ROS antagonizes Wnt signaling, an essential stimulus for osteoblastogenesis [11]. Importantly, *β*-catenin is not only a fundamental coactivator of FoxO3a for resistance to oxidative stress, but also a requisite mediator for downstream effector Tcf of canonical Wnt pathway, contributing to the regulation of bone mass [11, 18].

Antioxidants, generally used as healthy food or medicinal agents, have been suggested to have beneficial effects in oxidative stress-associated diseases. Recently, it was reported that endogenous antioxidant levels decreased to a low degree in osteoporosis patients, and vitamin C intake showed significant advantages in raising bone mineral density (BMD) [19]. To date, administration of antioxidant was ascertained to exhibit an inhibitory effect on ovariectomy-induced bone loss in rodent osteoporosis model [20]. Previous findings have demonstrated that *D*(+)*β*-3,4-dihydroxyphenyl lactic acid (Tanshinol, or named Danshensu) isolated from *Salvia miltiorrhiza* Bunge exerted the inhibitory influence on oxidative stress using *in vivo* and *in vitro* model systems [21–24]. The previous studies in our group indicated that Tanshinol protected bone from long-term use of excessive prednisone-induced bone marrow impairment by stimulating osteogenesis and depressing adipogenesis in bone marrow stromal cells (MSC) [25]. Furthermore, Tanshinol helps increase the expressions of Runx2 and *β*-catenin mRNA and decrease Dickkopf 1 (Dkk1) and Peroxisome Proliferator-activated receptor (PPAR*γ*) mRNA, which contributes to the positive impact on osteoblastic differentiation in rat MSC [26]. In addition, Resveratrol is a well-known antioxidant containing polyphenolic acid structure similar to Tanshinol (Figure 2(c)) and has been used as one of investigative tools for phytoestrogen to deal with osteoporosis [27–30]. The previous findings show that Resveratrol exerts protective effect on counteracting oxidative stress via the regulation of FoxO3a pathway [31, 32]. Hence, Tanshinol may abrogate the activation of FoxO3a signaling in response to oxidative stress, rescuing the dysregulation of Wnt signaling. However, evidence from the influence of Tanshinol on osteoblastic differentiation under oxidative stress and the underlying mechanisms remain to be elucidated. Additionally, Tanshinol, as principal active ingredient in many traditional Chinese medicine, has been widely used clinically for the treatment of cardiovascular diseases [33]. As a result, Tanshinol with advantages of clinical use may be developed as a potential candidate for prevention and/or treatment of osteoporosis.

Based on the arguments above, the aim of this study is to test the hypothesis that Tanshinol attenuates the deleterious effects of oxidative stress on osteoblastic differentiation in pluripotent mesenchymal precursor C2C12 cells and that the underlying mechanism may be involved in antagonizing the suppressing effects of oxidative stress via promoting the activation of canonical Wnt signaling and simultaneously abrogating the activation of FoxO3a pathway in C2C12 cells and preosteoblastic MC3T3-E1cells.

## 2. Materials and Methods 

### 2.1. Chemicals, Reagents, and Plasmids

Wnt3a and Dkk1 recombinant proteins were purchased from R&D Systems (Minneapolis, MN, USA). Recombinant human bone morphogenetic protein (BMP-2) was obtained from Peprotech (Rockyville, NJ, USA). Cignal FoxO3a reporter (FoxO3a-luc) and Cignal Tcf reporter (Tcf-luc) were purchased from Qiagen (Frederick, MD, USA). pcDNA3-*β*-catenin, pcDNA3-Tcf4, and pcDNA3 plasmid (empty vector) control were obtained from Guangzhou Promoter (Guangzhou, Guangdong, China). FoxO3a siRNA and the scrambled siRNA (scrambled) control were supplied by Shanghai GenePharma (Shanghai, China). Dual-luciferase reporter assay kit was purchased from Promega (Madison, WI, USA). ALP staining kit and Nuclear Protein Extraction Kit were obtained from Beyotime (Haimen, Jiangsu, China). Active caspase-3 ELISA kit and osteocalcin ELISA kit were purchased from Tuo Ke Da (Guangzhou, Guangdong, China). LY294002, *β*-catenin, FoxO3a, and *β*-actin antibodies were purchased from Cell Signaling Technologies (Boston, MA, USA). Histone H3 antibody was obtained from Santa cruz (Paso Robles, CA, USA). Hoechst 33258 and LiCl were purchased from Amresco (Solon, OH, USA). H_2_O_2_, MTT, Propidium iodide (PI), alizarin red S, and 2′,7′-dichlorodihydrofluorescein diacetate (DCFH-DA) were purchased from Sigma-Aldrich (St. Louis, MO, USA). Tanshinol and Resveratrol (positive control, abbreviated as Res.) were purchased from PI&PI (Guangzhou, Guangdong, China).

### 2.2. Cell Culture, Transfection, and Luciferase Activity

The pluripotent mesenchymal precursor C2C12 cells and preosteoblastic MC3T3-E1 cells were obtained from American Type Culture Collection (ATCC, Manassas, VA, USA) and cultured in Dulbecco's modified Eagle's medium (DMEM) and *α*-modified Eagle's medium (*α*-DMEM), respectively. Media were supplemented with 10% fetal bovine serum (FBS) and 1% each of penicillin, streptomycin, and glutamine and 1% sodium pyruvate. Cells were cotransfected transiently with indicated luciferase reporter constructs together with siRNA or overexpression constructs. Negative control was used with either scrambled RNA or empty vector plasmid. Cells were plated in 96-well plates at a density of 2 × 10^4^ cells/cm^2^ and transfected with a total of 0.3 *μ*g RNA and/or 0.2 *μ*g of DNA for 16 hours. Transfected cells were refreshed with fresh media and cultured for additional 8 hours. Subsequently, cells were serum starved by culturing in the presence of 2% FBS for 4 hours and treated with or without Tanshinol for 1 hour, followed by vehicle control (Con), H_2_O_2_, and/or related reagents, at indicated concentrations for 24 hours. Luciferase activity was measured using the Dual-Luciferase Reporter Assay System according to the manufacturer's instructions.

### 2.3. Cell Viability Assay

Cells were plated in their growth media in 96-well plates. After 24 hours of plating, cells were treated with indicated reagents and cultured sequentially for the indicated time period. After incubation with different treatments above, cells were cultured for further 4 hours at 37°C in refreshed media containing MTT (5 mg/mL). Then, the formazan crystals were solubilized in dimethyl sulfoxide (DMSO) and absorbance was measured at a wavelength of 570 nm using a microplate reader (Thermo Fisher Scientific, Vantaa, Finland).

### 2.4. Phenotypic Characterization of Osteoblastic Differentiation

C2C12 cells were seeded at a density of 2.4 × 10^4^ cells/cm^2^. At confluence, cells were cultured in osteogenic medium containing 5% FBS and BMP-2 (100 ng/mL) in the presence or absence of indicated reagents. The media were changed every 3-4 days. To identify committed osteoblasts, ALP staining was performed at day 3 using ALP Colour Development Kit. The stained cellular images were acquired by Eclipse E800 microscope (Nikon, Tokyo, Japan). For quantitative determination of ALP activity at day 3, cells were lysed in 100 mM glycine, 1 mM MgCl_2_, and 1% Triton X-100 at pH 10. Cell lysates were subjected to ALP activity using an ALP assay kit (Nanjing Jiancheng Biotech, China). Osteocalcin secretion in supernatants of the media at day 3 was measured using ELISA kit, as described in detail elsewhere [34]. Both ALP activity and osteocalcin concentration were normalized with total cellular protein contents, which were determined by a micro-Bradford assay. To measure mineralization activity, cells were stained with alizarin red S, as previously described [35]. Red staining was visualized and the representative pictures were photographed with the microscope mentioned above.

### 2.5. Oxidative Stress Assay

The intracellular oxidative stress was probed by DCFH-DA and quantified using flow cytometry. Briefly, cells were harvested by trypsinization and labeled with DCFH-DA (20 *μ*M) for 30 min at 37°C. Subsequently, cells were exposed to H_2_O_2_ in the presence or absence of Tanshinol or Resveratrol for 30 min, followed by fluorescence intensity acquirement using BD FACSCalibur flow cytometry (BD Biosciences, San Jose, CA, USA). Acquired data were analyzed with CellQuest Pro software (BD Biosciences, San Jose, CA, USA).

### 2.6. Cell Cycle Progression and Apoptosis Measurements

Cell cycle distributions were determined by flow cytometry to count cells stained with PI as described previously [36]. Morphological alteration of apoptotic cells was observed with Hoechst 33258 (20 *μ*M) [37], and fluorescent images were acquired by the microscopy mentioned above. Apoptotic cells characterized by nuclear damages and normal cells with round intact nuclear morphology were counted by Image J 1.46r software, respectively. Differences in ultrastructural morphology between normal cells and apoptotic cells were measured using transmission electron microscopy (TEM: H-7650, Hitachi, Tokyo, Japan), as described previously [38]. For cleaved caspase-3 assay, cells were harvested and lysed as described above. The activity of cleaved caspase-3 in cell lysates was determined using ELISA kit and normalized with total protein contents.

### 2.7. Quantitative RT-PCR Detection

RNA isolation and quantitative real-time polymerase chain reaction (qRT-PCR) were performed as described previously [39]. Briefly, total RNA from each indicated group was extracted by using TRIzol reagent. Complementary DNA (cDNA) was synthesized, and qRT-PCR was performed on a Stratagene Mx3005P QPCR System (La Jolla, CA, USA). The designed paired primers were as follows: *Gadd45a*, 5′-GGGCTCAGAGATGACTTTGC-3′ (forward), 5′-TTTTTGTCCCTTTTGCCTTG-3′ (reverse); *CAT*, 5′-CCTCGTTCAGGATGTGGTTT-3′ (forward), 5′-TCTGGTGATATCGTGGGTGA-3′ (reverse);* Axin2*, 5′-CTCCCCACCTTGAATGAAGA-3′ (forward), 5′-ACTGGGTCGCTTCTCTTGAA-3′ (reverse);* ALP*, 5′-AACCCAGACACAAGCATTCC-3′ (forward), 5′-GCCTTTGAGGTTTTTGGTCA-3′ (reverse);* OPG*, 5′-GCAGAAGGAACTGCAACACA-3′ (forward), 5′-ATGGTGAGGTGTGCAAATG-3′ (reverse); *GAPDH*, 5′-ATTGTCAGCAATGCATCCTG-3′ (forward), 5′-ATGGACTGTGGtcATGAGCC-3′ (reverse). PCR results were analyzed using Opticon Monitor Analysis 2.0 software (Bio-Rad Laboratories, Hercules, CA, USA). Relative mRNA expression was quantified by subtracting the glyceraldehyde 3-phosphate dehydrogenase (GAPDH) threshold cycle (*C*_*t*_) value from the *C*_*t*_ value of the genes of interest and expressed as 2^−Δ*C*_*t*_^, as described by the protocol of the manufacturer.

### 2.8. Western Blotting Analysis

For detection of *β*-catenin expression, cells were lysed by RIPA buffer. For measurement of FoxO3a protein, cells were homogenized for measurement of total FoxO3a and further extracted by nuclear extraction reagents for nuclear FoxO3a according to the instructions of the manufacturer, as described previously [40, 41]. The protein expression was monitored by the measurement of Chemiluminescence alterations using Image Station 2000 MM (Eastman Kodak, Rochester, NY, USA).

### 2.9. Statistical Analysis

ANOVA (SPSS 13.0) was used to detect effects of various treatments after establishing that the data were normally distributed and equivalency of variances. Samples were considered normally distributed if *P* > 0.05. Homogeneity of variance of the samples to be compared was tested by using a Levene's test. Heterogeneity of variance was accepted if *P* > 0.05, and LSD method was used to perform appropriate pairwise comparisons of treatment groups. Otherwise, Dunn's method for post hoc test was used to perform pairwise comparisons of treatment groups. Unless otherwise stated, results are presented as mean ± SEM and performed in triplicate and repeated at least one time.

## 3. Results

### 3.1. Tanshinol Ameliorates the Decrease of Cell Viability Induced by H_2_O_2_

**Figure 1:**
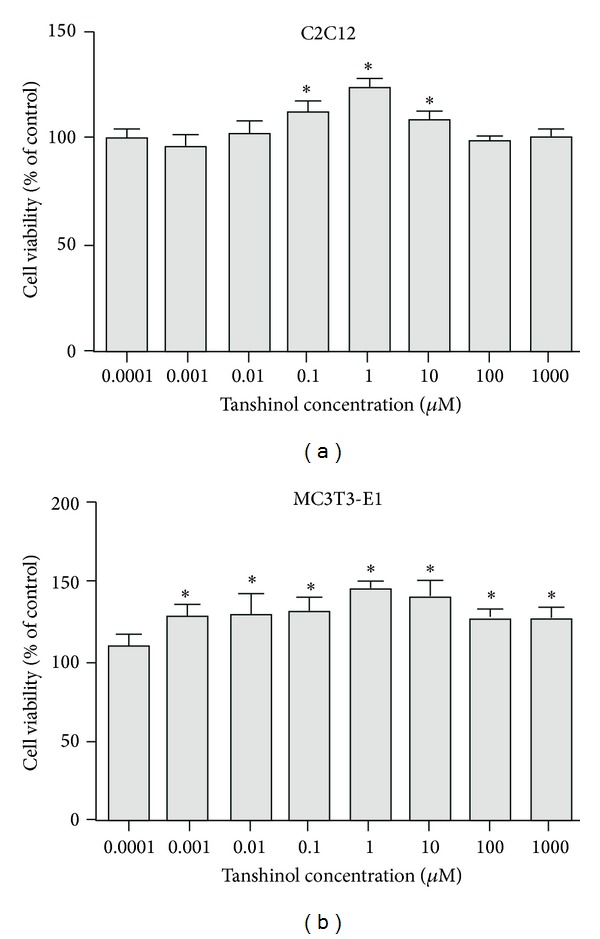
Effects of Tanshinol on cell viability of C2C12 cells and MC3T3-E1 cells. (a) Cell viability was measured by MTT assay in pluripotent mesenchymal precursor C2C12 cells and (b) preosteoblastic MC3T3-E1 cells treated with or without the indicated concentrations of Tanshinol for 24 h. Data are given as mean ± SEM of at least three independent experiments. *P < 0.05 versus vehicle control.

To test the effects of Tanshinol on cell growth, either C2C12 cells or MC3T3-E1 cells were exposed to Tanshinol in the tenfold increasing concentrations varied from 0.0001 to 1000 *μ*M for a period of 24 h. The results showed that Tanshinol with the experimental concentrations exerted no inhibitory effects on the growth of the two cells and that an increase in viability was observed in the two cell lines treated with Tanshinol, especially at the concentration of 1 *μ*M (Figures 1(a) and 1(b)).

**Figure 2:**
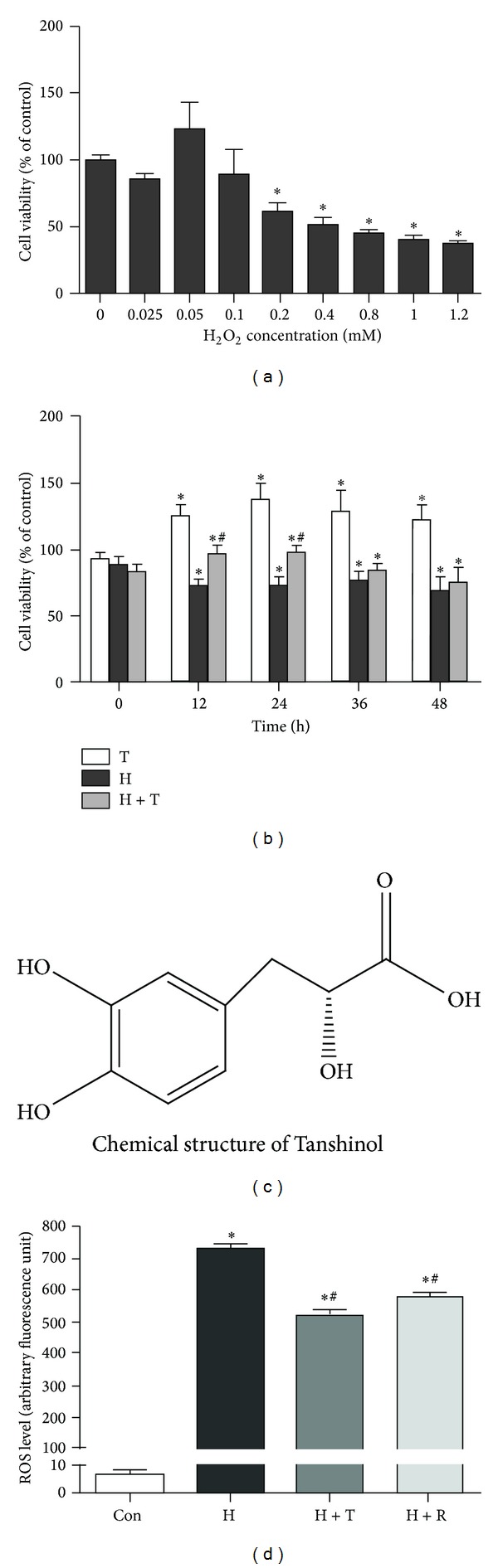
Protective effects of Tanshinol against H_2_O_2_-induced cell death and ROS generation. (a) Cell viability was measured by MTT assay in C2C12 cells exposed to H_2_O_2_ at the indicated concentration for 24 h. (b) C2C12 cells were pretreated in the presence or absence of Tanshinol (1 *μ*M) for 1 h before the addition of H_2_O_2_ (200 *μ*M) and were subsequently cultured for 0 h, 12 h, 24 h, 36 h, and 48 h, respectively. (c) The chemical structure of Tanshinol consists of polyphenolic hydroxyl groups. (d) C2C12 cells were pretreated as described in (b), followed by H_2_O_2_ (200 *μ*M) for 24 h, and the oxidative status of C2C12 cells was quantified by DCHF-DA to monitor the emitted fluorescence intensity resulting from intracellular oxidation using flow cytometry. Note: (1) Con (vehicle control); (2) H (H_2_O_2_); (3) H + T (H_2_O_2_ + Tanshinol); (4) H + R (H_2_O_2_ + Resveratrol). Data are given as mean ± SEM of at least three independent experiments. *P < 0.05 versus vehicle control and ^#^ *P* < 0.05 versus H_2_O_2_ treatment.

Cellular responses induced by H_2_O_2_ depend upon the severity of the cell damages, which are further influenced by magnitude of the dose. Therefore, we examined the viability of C2C12 cells treated with increasing concentrations of H_2_O_2_ ranging from 0.025 mM to 1.2 mM for a period of 24 h. As shown in Figure 2(a), cell viability showed a distinct trend toward reduction in a concentration-dependent manner in C2C12 cells treated with indicated concentration of H_2_O_2_ for 24 h, and there was a significant difference in high dose (0.2 mM or above) of H_2_O_2_. In contrast, cell viability showed no statistical alterations in C2C12 cells treated with H_2_O_2_ at low doses (0.1 mM or below). Next, the influence of Tanshinol (1 *μ*M) in preventing H_2_O_2_ (0.2 mM)-induced cell death was determined at different time points from 0 h, 12 h, 24 h, 36 h, to 48 h. It was obvious that 24-h treatment with 1 *μ*M Tanshinol could block the decrease of cell viability induced by H_2_O_2_ (Figure 2(b)). Based on these results, H_2_O_2_ at the dose of 0.2 mM and Tanshinol at the concentration of 1 *μ*M were adopted in the subsequent experiments.

### 3.2. Tanshinol Attenuates Oxidative Stress Triggered by H_2_O_2_

It is well known that intracellular ROS generation in response to oxidants stimuli contributes to a series of concomitant molecule events for oxidative stress. DCFH-DA is widely used as indicative of intracellular ROS generation [42]. To further examine whether Tanshinol with polyphenolic hydroxyl groups (Figure 2(c)) exerted an influence on suppression of cell death implicated in counteracting H_2_O_2_-induced oxidative stress, we measured intracellular ROS of C2C12 cells exposed to H_2_O_2_ (0.2 mM). Negligible fluorescence was observed in vehicle-treated C2C12 cells, whereas a remarkable increase of arbitrary fluorescence unit was detected in cells treated with H_2_O_2_ for 30 min, reaching an appropriately 7-fold higher than vehicle control. In contrast, treatment with either Tanshinol or resveratrol leads to the counteraction of ROS accumulation induced by H_2_O_2_ (Figure 2(d)). Additionally, the antioxidant effects of Tanshinol were indistinguishable from those of Resveratrol. Taken together, the results suggest that Tanshinol protects C2C12 cells against H_2_O_2_-induced oxidative stress by scavenging ROS generation.

### 3.3. Tanshinol Abrogates Inhibitory Effect of Oxidative Stress on Osteoblastic Differentiation

**Figure 3:**
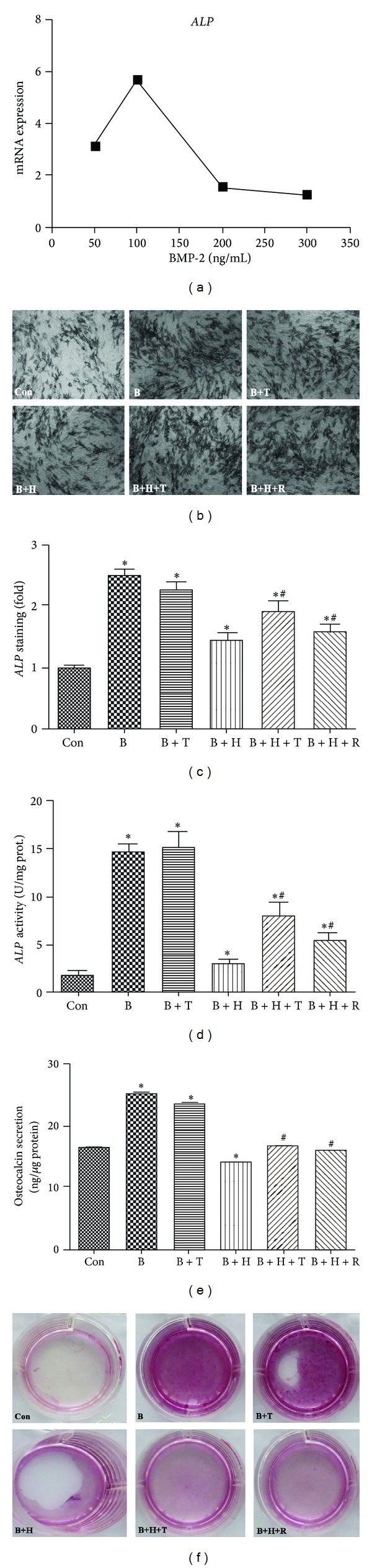
Tanshinol counteracts inhibitory effect of oxidative stress on osteoblastic differentiation. (a) C2C12 cells were treated with increasing concentrations of BMP-2 for 24 h to determine the optimal concentration using qRT-PCR. Cells were cotreated with BMP-2 (100 ng/mL) and Tanshinol (1 *μ*M) or Resveratrol (1 *μ*M) in the presence or absence of H_2_O_2_ (200 *μ*M), and determinations were made as follows. (b) Cells were stained using ALP staining Kit, and (c) the percentage of positively stained cell populations was counted by Image J software to analyse the extent of osteoblastic differentiation at day 3; (d) ALP activity in the cell lysates and (e) osteocalcin secretion in the supernatant of media were measured by ALP Assay Kit and ELISA Kit at day 3, respectively; (f) mineralization activity with the indicated treatments was stained using alizarin red S at day 10. Note: (1) Con (vehicle control); (2) B (BMP-2); (3) B + T (BMP-2 + Tanshinol); (4) B + H (BMP-2 + H_2_O_2_); (5) B + H + T (BMP-2 + H_2_O_2_ + Tanshinol); (6) B + H + R (BMP-2 + H_2_O_2_ + Resveratrol). Data shown are the mean ± SEM of at least three independent experiments. *P < 0.05 versus vehicle control and ^#^ *P* < 0.05 versus BMP-2 + H_2_O_2_ treatment. Original magnification ×100 in (b).

Considering that BMP-2 treatment diverts C2C12 cells from myogenic differentiation to the osteoblast lineage, we ascertained the optimal dose of BMP-2 (100 ng/mL) treatment with which the climax of *ALP* (a biomarker of osteoblastogenesis) mRNA expression was induced for 24 h (Figure 3(a)). To evaluate the capacity of Tanshinol or Resveratrol to protect C2C12 cells against oxidative stress in the process of osteoblastic differentiation, we performed short-term osteoblast differentiation experiments. ALP staining and its intensity quantification indicated that, in comparison to vehicle control, both Tanshinol and Resveratrol display a significantly inhibitory influence on the decrease of cell populations stained positive for ALP under oxidative stress (Figures 3(b) and 3(c)). We next verified that Tanshinol could rescue the secreted osteocalcin in the supernatant of media and ALP level in the cell lysates in C2C12 cells simulated by BMP-2 at day 3 under oxidative stress, as well as Resveratrol (Figures 3(d) and 3(e)). Additionally, C2C12 cells committed to osteoblast were investigated by alizarin red S staining after long-term cultures to depict mineralization of the osteoblast nodules. The extent of alizarin red S staining decreased significantly in C2C12 cells exposed to H_2_O_2_ at day 10, and either Tanshinol or Resveratrol reversed the antagonistic effect of H_2_O_2_ on the osteogenic capacity (Figure 3(f)). Taken together, these lines of evidence in our experiments indicate that Tanshinol may counteract the deleterious effect of H_2_O_2_-elicited oxidative stress on BMP-2 inducible osteoblastic differentiation.

### 3.4. Tanshinol Protects against H_2_O_2_-Induced Cell Cycle Arrest and Apoptosis

**Figure 4:**
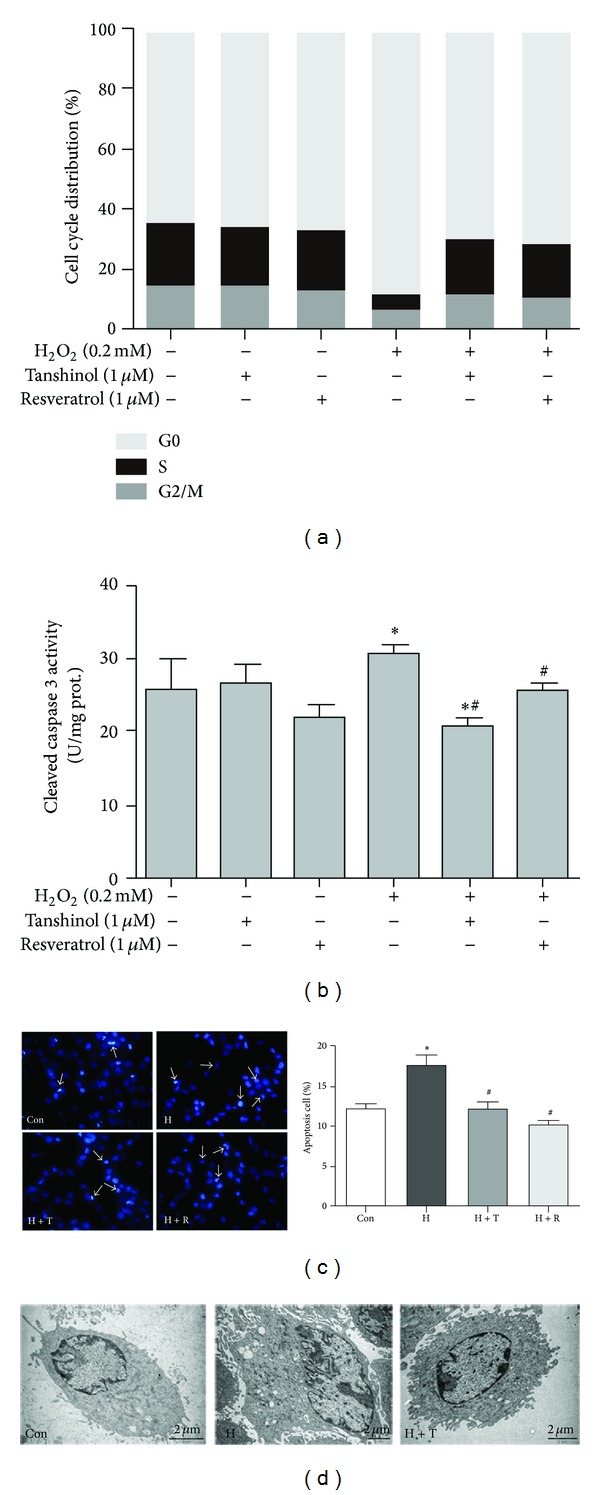
Tanshinol diminishes the cell cycle arrest and apoptosis under oxidative stress. C2C12 cells were pretreated with the indicated concentrations of Tanshinol or Resveratrol for 1 h, followed by the treatment of vehicle control or H_2_O_2_ for 24 h. The following measurements were carried out. (a) Analysis of cell cycle distribution was explored using flow cytometry; (b) the activation of caspase 3 was determined by ELISA kit; (c) observations of morphological alterations of apoptosis cells were addressed by Hoechst33258 staining (left panel) and counted by Image J software (right panel); (d) the ultrastructural differences between apoptosis cells and normal cells were examined using TEM. Note: (1) Con (vehicle control); (2) H (H_2_O_2_); (3) H + T (H_2_O_2_ + Tanshinol); (4) H + R (H_2_O_2_ + Resveratrol). Error bars indicate mean ± SEM of at least three independent experiments. *P < 0.05 versus vehicle control and ^#^ *P* < 0.05 versus H_2_O_2_ treatment. Original magnification ×400 in (c) (left panel).

Encouraged by the findings above, we proceeded to examine whether the decreased capacity of proliferation and differentiation elicited by oxidative stress is relevant to cell cycle arrest and apoptosis. To begin with, a sharp increase of the cell proportion in G0 phase was observed in C2C12 cells exposed to H_2_O_2_, as well as a corresponding decrease of cells population in the both S phase and G2/M phase. Encouragingly, Tanshinol showed a capacity to reverse the cell cycle arrest at S and G2/M phases, as effectively as Resveratrol, whereas C2C12 cells treated with either Tanshinol or Resveratrol alone had no apparent influence on cell cycle progression (Figure 4(a)).

We next evaluated the changes of apoptosis morphology in the chromatin structure of C2C12 cells using Hoechst33258 staining. The results showed that C2C12 cells underwent apoptosis characterized by chromatin condensation, apoptotic bodies, and nuclear enlarged or fragments after treatments with H_2_O_2_ for 12 h (Figure 4(c)). These morphological criteria of apoptotic alterations were further confirmed by ultrastructure analysis using TEM assay. A clearly apoptotic alteration occurred in C2C12 cells stimulated by oxidative stress with characteristics of nuclear protrusion and swelling, cytoplasmic vacuoles, chromatin margination, and membrane disintegration (Figure 4(d)). Meanwhile, pretreatment for 12 h with Tanshinol or Resveratrol reduced the extent of apoptosis in C2C12 cells exposed to H_2_O_2_ (Figures 4(c) and 4(d)). Caspase-3 is required for some typical hallmarks of apoptosis and is indispensable for cell death in a caspase-3-dependent manner that active Caspase-3 executes eventually cell death program [2]. Therefore, we further ascertained that the activity of caspase-3 cleavage in H_2_O_2_-treated cells was higher than that in cells exposed to vehicle control, and Tanshinol could suppress the deleterious influence (Figure 4(b)). Collectively, all these lines of evidence provide further support that Tanshinol is capable of abrogating apoptosis of C2C12 cells triggered by oxidative stress.

### 3.5. Tanshinol Reverses Inhibition of Wnt/*β*-Catenin Signaling Pathway under Conditions of Oxidative Stress

**Figure 5:**
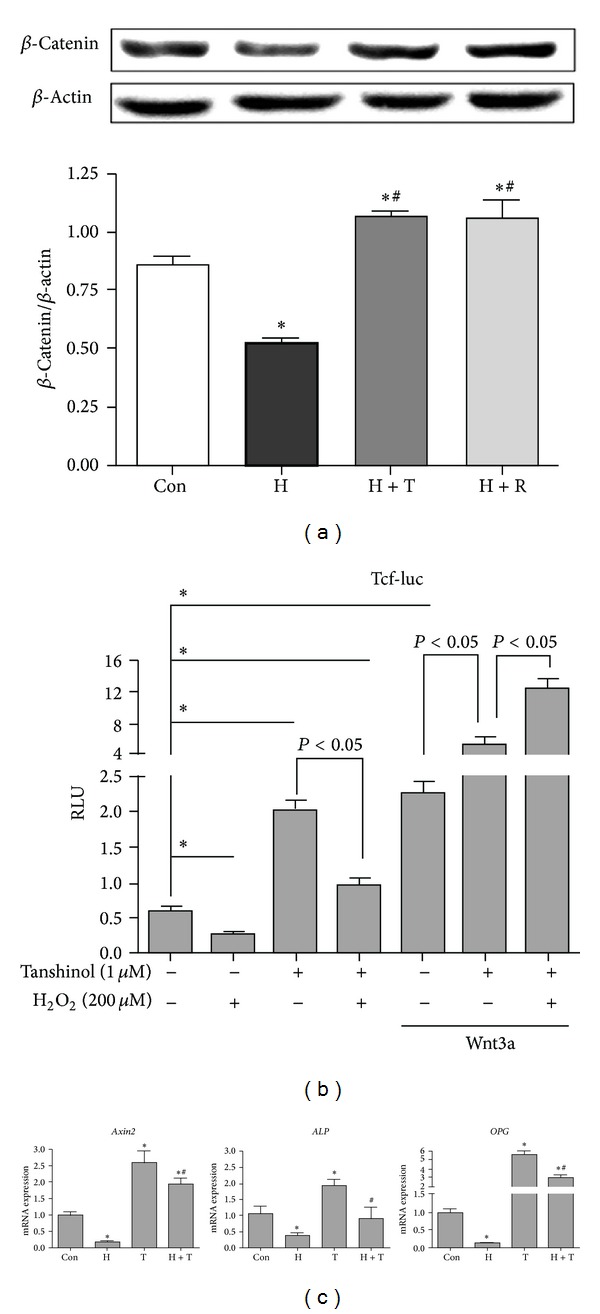
Tanshinol rescues oxidative stress-elicited inhibition of Wnt/*β*-catenin signaling. (a) C2C12 cells were treated as described in Figure 4, and protein levels of *β*-catenin were detected by Western Blot. Representative immunoblots were shown in upper panel. Quantitative results of relative band intensities of protein are showed in lower panel. (b) C2C12 cells were transfected with the Tcf-luc reporter plasmid or negative control. Cells transfected were treated with or without Tanshinol in the presence or absence of Wnt3a for 1 h, followed by vehicle control, H_2_O_2_ for 24 h. Luciferase activity assays were explored as described under “Section 2”. The data represent mean ± SEM of luciferase relative luminescence units (RLU) normalized to corresponding *Renilla* luciferase activity (triplicates). (c) C2C12 cells were treated as described in Figure 4, and the expression levels of *Axin2*, *ALP*, and *OPG* mRNA were quantified by qRT-PCR and normalized to *GADPH* mRNA. Note: (1) Con (vehicle control); (2) H (H_2_O_2_); (3) T (Tanshinol); (4) H + T (H_2_O_2_ + Tanshinol); (5) H + R (H_2_O_2_ + Resveratrol). Error bars indicate mean ± SEM of at least three independent experiments. *P < 0.05 versus vehicle control and ^#^ *P* < 0.05 versus H_2_O_2_ treatment.

Based on the evidence mentioned above, we next asked whether Tanshinol promoted activation of Wnt signaling pathway required for osteoblast commitment under conditions of oxidative stress. Using Western Blot, we consolidated that the decrease of *β*-catenin (a key molecular of canonical Wnt signal transduction) in C2C12 cells exposed to H_2_O_2_ was restored by the treatment of Tanshinol, just like Resveratrol (Figure 5(a)).

To elucidate the role of Tanshinol in upregulation of Wnt pathway under the condition of oxidative stress, we chose a direct measure to monitor Wnt signaling through alterations of Tcf-mediated transcription using a Tcf-luc reporter plasmid. As expected, the luciferase relative luminescence units (RLU) of Tcf-luc in C2C12 cells treated with H_2_O_2_ were significantly declined to the extent of approximate half of the RLU in cells treated with vehicle control, and Tanshinol attenuated the decrease of RLU caused by oxidative stress. Moreover, Tanshinol treatment alone enhanced the increase of Tcf-luc activity, as effectively as treatment with Wnt3a, showing a more than 4-fold increase of RLU. Importantly, Tcf transcriptional activity stimulated by Tanshinol in combination with Wnt3a achieves a dramatically higher level (as great as tens of times of vehicle control) under conditions of oxidative stress than that in normal conditions, exhibiting an approximate 10-fold increase compared with vehicle control (Figure 5(b)).

To further test whether Tanshinol ameliorates the downregulation of Wnt signaling transduction under oxidative stress, we examined the mRNA expression levels of target genes of Wnt signaling in C2C12 cells in response to Tanshinol under oxidative stress, using quantitative RT-PCR. As expectedly, the mRNA expression levels of *Axin2*, *ALP* and *OPG* genes were hampered by H_2_O_2_ treatment, and Tanshinol antagonized the inhibitory impact of H_2_O_2_ on the activation of these Wnt target genes (Figure 5(c)). In brief, these lines of evidence confirm that Tanshinol reverses the inhibition of Wnt signaling under oxidative stress.

### 3.6. Tanshinol Blocks the Activation of FoxO3a Signaling in response to Oxidative Stress

**Figure 6:**
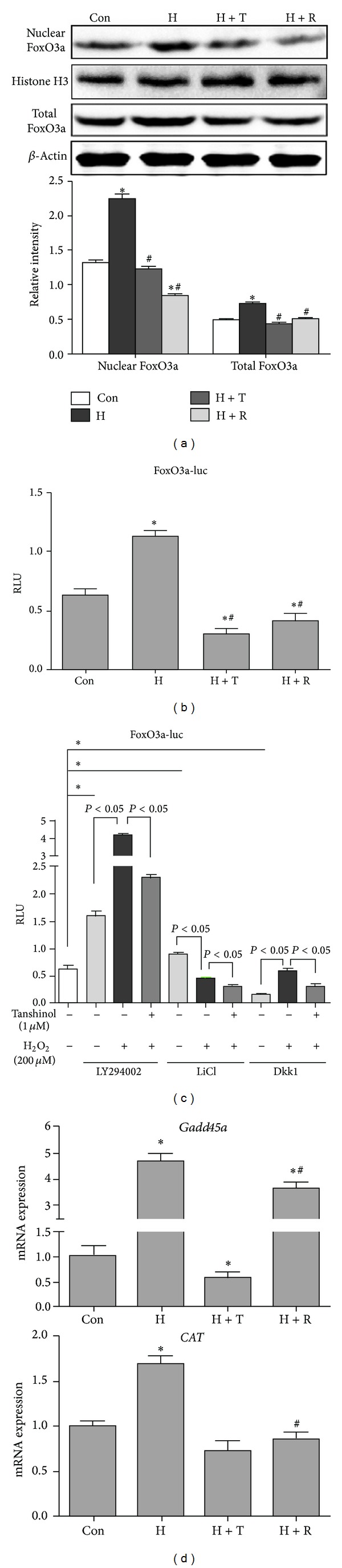
Tanshinol attenuates the activation of FoxO3a in response to oxidative stress. (a) C2C12 cells were treated as described in Figure 4, and protein expressions from whole cell lysates and nuclear fractions were immunoblotted for FoxO3a, *β*-actin (whole lysates marker), and Histone H3 (nuclear marker). Representative immunoblots were shown in upper panel. Quantitative analysis of relative band intensities of protein was showed in lower panel. (b and c) Cells were transfected with the FoxO3a-luc reporter plasmid, and the treatment of Wnt3a was replaced with LY294002 (50 *μ*M), LiCl (10 mM), or Dkk1 (500 ng/mL), and other procedures of experiments were addressed as described in Figure 5(b). (d) C2C12 cells were treated as described in Figure 4, and the expression levels of *Gadd45* and *CAT* mRNA were quantified by qRT-PCR and normalized to *GADPH* mRNA. Note: (1) Con (vehicle control); (2) H (H_2_O_2_); (3) T (Tanshinol); (4) H + T (H_2_O_2_ + Tanshinol); (5) H + R (H_2_O_2_ + Resveratrol). Error bars indicate mean ± SEM of at least three independent experiments. *P < 0.05 versus vehicle control and ^#^ *P* < 0.05 versus H_2_O_2_ treatment.

We next asked whether Tanshinol inhibited the activation of FoxO3a-mediated transcription in response to oxidative stress. Firstly, using Western Blot, we proceeded to validate that both total FoxO3a and nuclear FoxO3a protein were activated in C2C12 cells treated with H_2_O_2_ over a 24 hour period compared with vehicle control and that Tanshinol or Resveratrol could diminish the increase of expression levels of FoxO3a (Figure 6(a)). Subsequently, the capacity of Tanshinol to regulate FoxO3a pathway was monitored by using FoxO3a-luc reporter construct. While H_2_O_2_ stimulation is responsible for the increase of FoxO3a-luc, in line with the evidence previously mentioned [11], Tanshinol treatment alone exerted an inhibitory effect on the RLU of FoxO3a-luc for approximate one-half reduction compared with vehicle control, and counteracted the increase of FoxO3a-luc evoked by H_2_O_2_ in C2C12 cells (Figure 6(b)). Furthermore, FoxO3a is one of the important downstream targets of the PI3K/Akt pathway in relevance to oxidative stress [43]. We further substantiated that the transcription activity of FoxO3a-luc was significantly induced by treatment with specific PI3K inhibitor LY294002 in C2C12 cells in the presence or absence of H_2_O_2_, and Tanshinol could block the activation of FoxO3a transcription activity triggered by LY294002 under the condition of oxidative stress.

Notwithstanding as for the putative role for the cross-talk between Canonical Wnt signaling and FoxO3a pathway under oxidative stress, we modulated *β*-catenin levels chemically and further examined the influence of Tanshinol on FoxO3a-luc activity. C2C12 cells are pretreated with either GSK-3*β* inhibitor LiCl to mimic Wnt signaling by inducing the accumulation of *β*-catenin, or with LRP5/LRP6 inhibitor Dkk1 protein to block Wnt signaling transduction, respectively. Consistent with previous reports [11, 18], treatment with LiCl enhanced FoxO3a activity in C2C12 cells, while cells treated with Dkk1 showed an inhibitory effect on FoxO3a signaling (Figure 6(c)). Conversely, the increase of H_2_O_2_-stimulated RLU of FoxO3a-luc activity was abolished by Tanshinol regardless of blockade or activation of Wnt signaling, just as treatment with Tanshinol alone under oxidative stress (Figure 6(c)).

Based on the regulation of Tanshinol on the activation of FoxO3a transcription activity in response to oxidative stress, we ascertained the induction of mRNA expression levels of FoxO3a target genes such as *Gadd45* and *CAT* were hindered by Tanshinol in C2C12 cells under oxidative stress using qRT-PCR (Figure 6(d)). In brief, the inhibitory role of Tanshinol on activation of FoxO3a pathway may be responsible for suppression of oxidative stress and involved in regulation of Wnt signaling.

### 3.7. Alleviation of Oxidative Stress-Elicited Wnt/Tcf Inhibition by Tanshinol Is Mediated via FoxO3a

**Figure 7:**
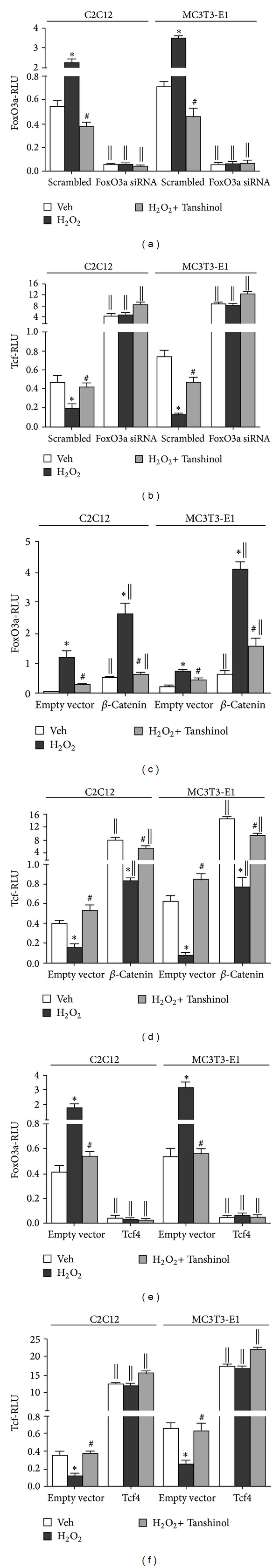
Effects of Tanshinol on Tcf- and FoxO3a-mediated transcription activity in C2C12 cells and MC3T3-E1 cells treated with FoxO3a siRNA, or with overexpression of *β*-Catenin or Tcf4 under oxidative stress. ((a), (c), and (e)) C2C12 cells or MC3T3-E1 cells were cotransfected with the FoxO3a-luc reporter plasmid in combination with FoxO3a siRNA, or pcDNA3-*β*-catenin, or pcDNA3-Tcf4, or corresponding control using Attractene transfection reagent. Other procedures were carried out as described in Figure 5(b). ((b), (d), and (f)) Cells were cotransfected with Tcf-luc reporter plasmid and other treatment described in (a), (c), and (e). Others procedure were made as described in Figure 5(b). Bars indicate mean ± SME of triplicate determinations. *P < 0.05 versus vehicle control and ^#^ *P* < 0.05 versus H_2_O_2_ treatment; ^||^ *P* < 0.05 versus corresponding empty vector or scrambled control.

**Figure 8:**
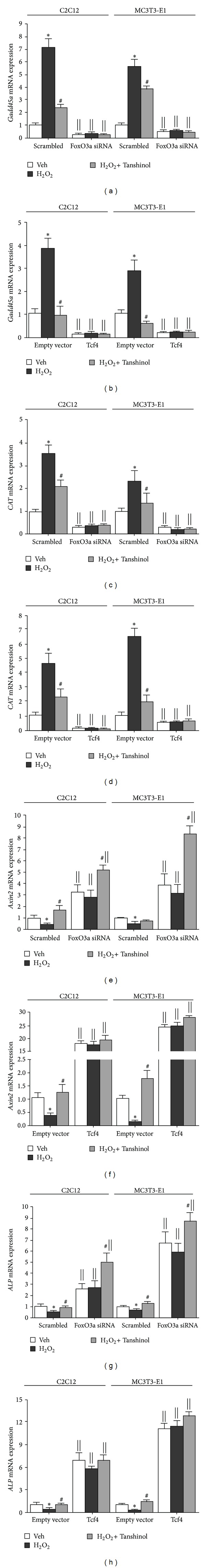
Effects of Tanshinol on targets genes of Wnt/Tcf and FoxO3a in C2C12 cells and MC3T3-E1 cells treated with FoxO3a siRNA, or with Tcf4 overexpression under oxidative stress. ((a), (c), (e), and (g)) C2C12 cells or MC3T3-E1 cells were transfected transiently with either the FoxO3a siRNA or the scrambled RNA control in the presence or absence of vehicle, H_2_O_2_, or Tashinol for 24 hours. The relative mRNA expression levels of *Gadd45a*, *CAT*, *Axin2*, and *ALP* genes in control cells (Scrambled) and in the FoxO3a-knockdown (FoxO3a siRNA) cells were assessed by quantitative RT-PCR assay. ((b), (d), (f), and (h)) C2C12 cells or MC3T3-E1 cells were transfected transiently with either the overexpression plasmid of Tcf4 or the empty vector control. The following procedure was executed as described in (a), (c), (e), and (g). mRNA values were normalized to GAPDH mRNA. Bars represent mean ± SME of three independent experiments. *P < 0.05 versus vehicle control and ^#^ *P* < 0.05 versus H_2_O_2_ treatment; ^||^ *P* < 0.05 versus corresponding empty vector or scrambled control.

Having elucidated the inhibitory roles of Tanshinol on activation of FoxO-mediated transcription involved in regulation of the canonical Wnt signaling under oxidative stress, we examined more directly whether overexpression of *β*-catenin or knockdown of FoxO3a with siRNA interference could augment the influence of Tanshinol on antioxidative stress in C2C12 cells and MC3T3-E1 cells. Indeed, transient knockdown of FoxO3a in the two cell lines exerted a reduction of FoxO3a transcription activity and a concomitant decrease in the mRNA expressions levels of FoxO3a target genes like* Gadd45a* and *CAT* in the presence or absence of H_2_O_2_ treatment (Figures 7(a), 8(a), and 8(c)). Contrarily, activation of Tcf transcription factor and mRNA expression levels of Wnt target genes, such as *Axin2* and *ALP*, showed strikingly an increase owing to knockdown of FoxO3a, and no alterations in Wnt signaling were observed in cells treated with or without H_2_O_2_. Encouragingly, the protective role of Tanshinol on Wnt pathway seemed more strong in cells when transfected with FoxO3a siRNA than that in cells transfected with scrambled RNA, especially in conditions of oxidative stress (Figures 7(b), 8(e) and 8(g)).

Having confirmed the inhibitory effect of Tanshinol on activation of FoxO3a activity in C2C12 cells treated with LiCl, we next investigated whether the activation of canonical Wnt signaling by way of *β*-catenin overexpression hampered FoxO-mediated transcription. As shown in Figures 7(c) and 7(d), a similar increasing trend in both Tcf- and FoxO3a-mediated transcription activity was observed in the two cell lines treated with pcDNA3-*β*-catenin. Meanwhile, Tanshinol counteracted the decrease of Tcf transcription activity and simultaneously alleviated the increase of FoxO3a transcription activity. The previous evidence indicated that FoxO and Tcf4 compete for interaction with *β*-catenin and that the interaction of FoxO with *β*-catenin inhibits Wnt/Tcf pathway [44]. Then, we confirmed that the transcription activity of FoxO3a was reduced to a lower level in cells treated with pcDNA3-Tcf4; in contrast, Wnt/Tcf signaling was activated by overexpression of Tcf4 in C2C12 cells and MC3T3-E1 cells treated with or without H_2_O_2_ (Figures 7(e), 7(f), 8(b), 8(d), 8(f), and 8(h)). Taken together, these findings provide evidence that Tanshinol rescues the inhibition of Wnt/*β*-catenin/Tcf signaling under oxidative stress through downregulation of FoxO3a transcription activity in C2C12 cells and MC3T3-E1.

## 4. Discussion

In skeletal tissue, excessive accumulation of ROS and the subsequent activation of oxidative stress play contributory roles in the development and progression of osteoporosis [45, 46]. Based on well-documented evidence highlighting the role of ROS production in age-related osteoporosis or in secondary osteoporosis, we confirmed herein that oxidative stress diminished osteoblastic differentiation and this might be rescued by antioxidants. Previously, we showed that a natural antioxidant of Tanshinol has the potential to promote osteoblastic differentiation, bone matrix formation, and bone mineralization and thus shows a strongly protective effect to counteract Glucocorticoid (GC)-induced osteoporosis [25], as efficiently as Resveratrol [29, 30, 47–49]. The evidence presented in this report illustrates that Tanshinol ameliorates the deleterious effects of oxidative stress on the proliferation and BMP-2-stimulated osteoblastic differentiation in C2C12 cell line, a pluripotent mesenchymal precursor (Figures 2 and 3). Furthermore, we observed that the protective effect of Tanshinol is linked to relief of cell cycle arrest and counteraction of apoptosis (Figure 4). Specially, we extended the work in this study to confirmation of the protective impact of Tanshinol on osteoblastic differentiation function under conditions of oxidative stress via activation of Wnt signaling cascade and to elucidation of the capacity of Tanshinol to cope with oxidative stress through suppression of FoxO3a transcription factor (Figures 5, 6, 7, and 8).

Tanshinol is one of polyphenolic acid components isolated from *Salvia miltiorrhiza* Bunge, and its chemical structure consists of several polyphenolic hydroxyl groups (Figure 2(c)), just like Resveratrol [50], which contributes to the suppression of oxidative stress via regulation of FoxO3a transcription factor [31, 32, 51]. What are the underlying mechanisms whereby Tanshinol protects against oxidative stress in the process of osteoblastic differentiation? Based on the results of the present study and previous reports, there are at least several plausible mechanisms. To begin with, the findings from our study show that H_2_O_2_ provokes the increase in oxidative stress and subsequently resulted in the decrease in cell viability, which is consistent with previous work of the others (Figure 2) [52]. Moreover, the further evidence presented in this report demonstrates that the apoptotic response is observed morphologically and the activation of caspase-3 reveals an underlying mechanism of cell death in a caspase-3-dependent manner during the process of apoptosis (Figure 3). As is well known, the extrinsic and intrinsic pathways of apoptosis principally converge in the caspase cascade by the activation of downstream effector caspase-3, which is initiated by oxidative stress to transform into cleaved caspase-3 and then exert the effect in the last stage of cell death, including cellular shrinkage, cellular membrane phosphatidylserine (PS) exposure, DNA fragmentation, and eventually apoptosis [53, 54]. Additionally, active caspase-3 has the capacity to cleave Akt, leading to the inhibition of the PI3K/Akt signaling pathway, which in turn relocates FoxOs proteins back into the nucleus to activate transcriptional activity and induces simultaneously cell cycle arrest and apoptosis [55, 56]. Briefly, our data suggest a molecular mechanism that the preventive effects of Tanshinol on cell cycle arrest and apoptosis induction initiated by ROS may be associated with regulation of the caspase-3/FoxO3a pathway under oxidative stress. Future investigation is needed to address the question of how Tanshinol downregulates caspase-3/FoxO3a pathway cascade to abrogate the activation of apoptosis, which helps to shed light on our understanding of the protective role of Tanshinol on osteoblastic differentiation under conditions of activation of FoxO3a in cellular responses to oxidative stress.

The evidence in the present study indicates that Tanshinol contributes to the suppression of the deleterious impacts of oxidative stress on osteoblastic differentiation in C2C12 cells stimulated by BMP-2. Previous studies have shown that BMPs comprise a subfamily within the TGF-*β* superfamily and exert an important role in skeletal development and adult bone homeostasis [57]. Consistent with the previous findings [58], continuous BMP-2 exposure converts the differentiation switch of C2C12 myoblasts into osteoblast lineage cells (Figure 3). Furthermore, treatment with Tanshinol ameliorates the decline of osteogenic differentiation in C2C12 cells stimulated by BMP-2 under oxidative stress. Evidence of *in vitro* studies previously implied that *β*-catenin is a required downstream factor of BMP-2 for osteoblast mineralization by the way of a Wnt autocrine loop [59]. Our findings indicate that treatment with Tanshinol in C2C12 cells contributes to the upregulation of Wnt signaling. Taken together, the findings in this study suggest that Tanshinol may be conducive to osteoblastic differentiation under oxidative stress via Wnt/BMP-2 pathway cascade.

Although it is well accepted that oxidative stress stimulates the activation of FoxO3a signaling pathway and subsequently leads to its competition with Tcf protein for the association with the limited pool of *β*-catenin, the interactions between oxidative stress and the Wnt signaling pathway remain controversial. Several lines of evidence support the functional role of oxidative stress in the downregulation of Wnt pathway in A14, C2C12, OB-6, and/or HEK293 cells [11, 18, 60], other researchers, however, have an opposite notion that oxidative stress contributes to activation of the Wnt/*β*-catenin pathway and downstream Tcf signaling in L cells, NIH3T3 or HEK293 cells [61], endothelial cells [62], and a rat model of diabetic retinopathy [63, 64]. Herein, different from the evidence mentioned above, results from our experiments in C2C12 cells and MC3T3-E1 cells have supported that Wnt pathway downstream Tcf signaling is suppressed by oxidative stress, and antioxidant Tanshinol reverses the dysregulation of Tcf transcription activities. The finding is similar to our previously established work that Tanshinol inhibits Dkk1 mRNA level and stimulates the expression of *β*-catenin and Runx2 in the process of rat marrow stromal cell differentiation with or without GC treatment [26]. However, Tcf signaling pathway is activated dramatically by oxidative stress in C2C12 cells in the presence of a combination of Wnt3a and Tanshinol, approximately 4-fold higher than that without oxidative stress. It may be that Tanshinol upregulates Wnt pathway cascade synergistically with Wnt3a to ameliorate deleterious effects of oxidant on the function of cells under oxidative stress.

The discrepancy between the results of this opposite evidence and ours may be due to the different experimental design and may be lie in different mechanisms. One causative role in the positive effect of oxidative stress in the presence of exogenous Wnt3a on the activation of Wnt signaling may be associated with endogenous nucleoredoxin (NRX), which acts as a suppressor of Wnt/*β*-catenin signaling by the association with disheveled (Dvl), and the complex between Dvl and NRX is impaired feasibly by the oxidation like H_2_O_2_ treatment, leading to the activation of Wnt signal cascade from receptor Frizzled to Tcf transcription factor [61]. Another reason of the activation of Wnt pathway under oxidative stress may originate from the stabilization of Wnt receptor of LRP6 and the increase of LRP6 level caused by oxidant, such as H_2_O_2_ [64].

Eventually, the evidence indicated that either FoxO3a siRNA or overexpression of Tcf4 shows the similar influence on the activation of Wnt signaling and suppression of FoxO3a transcription activity in C2C12 cells and MC3T3-E1 cells treated with or without oxidant stimulus. Both Tcf- and FoxO3a-mediated transcription activities, however, are elevated by virtue of overexpression of *β*-catenin in cells under oxidative stress. As reported previously [18, 44], it is vital for cells to diverge from Tcf to FoxO3a under conditions of oxidative stress, and a role for *β*-catenin in the regulation of resistance against oxidative stress has been highlighted in these studies. There is a disagreement between the previous evidence and the observations reported here. A more intriguing and possible explanation for this difference could reside within the present fact that it is not *β*-catenin protein for counteraction the activation of FoxO3a transcription factor under oxidative stress, but Tcf transcription factor. Therefore, the suppression of activation of FoxO3a contributes to antagonizing inhibitory effect of oxidative stress on Wnt/Tcf pathway that may be dependent on the competency of Wnt signaling. Interestingly, it seems that Tanshinol exerts no effect on FoxO3a activity or Wnt signaling in cells under conditions of FoxO3a siRNA interference or Tcf4 overexpression. Meanwhile, a dual ameliorating influence of Tanshinol on activation of FoxO3a-mediated transcription and inhibition of Tcf-mediated transcription is observed in cells under oxidative stress. Further research should be required to address and expand the present findings *in vivo*.

In summary, we have provided herein a novel feasible molecular explanation of Tanshinol in counteracting the inhibitory effect of oxidative stress on osteoblastic differentiation. These results strongly support the viewpoint that Tanshinol scavenges ROS and subsequently attenuates oxidative stress, resulting in downregulation of FoxO3a signal responsible for caspase-3-dependent apoptosis and cell cycle arrest. Moreover, Tanshinol upregulates Wnt signal cascade that is achieved by increasing activity of Tcf transcription factors for osteoblastic differentiation under oxidative stress. Our findings suggest that the beneficial effects of Tanshinol may be developed as a novel therapeutic approach in recently recognized conditions of niche targeting osteoporosis. Actually, Tanshinol used as a principal active ingredient of approved drug for the treatment of cardiovascular diseases for ten years in China, further studies will be needed to investigate whether it may be a candidate for prevention and therapeutic applications in osteoporosis through manipulation of the Wnt/FoxO3a pathway in bone tissue.
